# Ti-Al-V/Zn-Al-Cu Composite Materials Prepared by Zinc Melt Infiltration Technology

**DOI:** 10.3390/ma18204690

**Published:** 2025-10-13

**Authors:** Veronika Balejová, Alena Michalcová, Martina Bašistová, Petr Lichý, Dalibor Vojtěch

**Affiliations:** 1 Department of Metals and Corrosion Engineering, University of Chemistry and Technology, Prague, Technická 5, 166 28 Prague, Czech Republic; balejovv@vscht.cz (V.B.); vojtechd@vscht.cz (D.V.); 2 Department of Metallurgical Technologies, Faculty of Materials Science and Technology, VŠB-Technical University of Ostrava, 17. Listopadu 2172/15, Poruba, 708 00 Ostrava, Czech Republic; martina.basistova@vsb.cz (M.B.); petr.lichy@vsb.cz (P.L.)

**Keywords:** zinc, Ti-6Al-4V, centrifugal casting, biomaterial, selective laser melting

## Abstract

This work deals with the preparation and characterization of TiAlV/ZnAlCu composite materials. The aim is to create a model for biomaterial with good biocompatibility and acceptable mechanical properties. Infiltrating zinc into the reinforcement made of the titanium alloy could significantly improve the osseointegration of the bioimplant made from this material. The investigated reinforcements of three different geometries made from Ti-6Al-4V prepared by the SLM method (selective laser melting) were infiltrated with molten zinc or the Zn-based alloy. Two infiltration approaches were used—suction of the melt using a vacuum pump and centrifugal casting. By these procedures, different infiltration rates were achieved. Furthermore, the mechanical properties of the prepared composite materials were characterized by compression tests. The results were compared with the mechanical properties of the Ti-6Al-4V alloy reinforcement.

## 1. Introduction

Titanium and its alloys are widely used to make implants [1]. They are distinguished by their corrosion resistance, low density and biocompatibility [2]. The only disadvantage is that their strength is too high, which can cause a stress-shielding effect [3]. This can be reduced by using a special porous design, which offers another area of research—a biogenic element can be infiltrated into the pores, which could further improve biocompatibility and the ability to osseointegrate [4]. The organism would gradually absorb the biogenic element and instead build new tissue that would fit well on the implant [5,6]. Zinc is a suitable biogenic element for several reasons—it is involved in a number of metabolic pathways of various enzymes that are essential for the human body, and its antimicrobial effects are also important [7,8,9]. The alloy system that forms different phases of zinc with titanium is quite complex and it is still under investigation [10]. Its full exploration is crucial for the preparation of new biocompatible alloys. Understanding the kinetics of the formation of the intermetallic phases is also essential for designing a suitable production process. Indeed, it has been shown that with prolonged contact between titanium and zinc, the two metals react together to form an undesirable layer of brittle intermetallics, the formation of which must be prevented [11].

A method that could provide a sufficiently high melt cooling rate could be the centrifugal casting method. In addition, unlike conventional casting methods, it provides higher quality and more accurate castings [12]. Nowadays, the trend in metallurgy is towards additive methods of producing metallic materials. Additive methods are exclusive especially in their ability to create geometrically complex structures that would be difficult to create by other methods [13,14]. With these methods, each patient could receive special care, and it would not be a problem to produce a tailor-made implant for them in a short time [15].

The disadvantages of additive methods are the defects created during printing. The most common are gas pores or keyhole pores, as well as areas of insufficient fusion and balling phenomenon on the surface of the material [16,17]. These defects have to be taken into account in the development process of the material.

The recently studied Ti/(Mg-)Zn composites are usually prepared by friction stir processing [18,19] of gravity casting [20]. We have chosen the process of infiltration of the Zn (-based) melt into Ti-6Al-4V 3D-printed scaffold serving as reinforcement. The conditions used for additive manufacturing of Ti-6Al-4V by selective laser malting (SLM) technology were described in [21]. The Ti-6Al-4V alloy was taken as a most typical 3D-printed Ti-based alloy. For the same reason, the Zn-4Al-3Cu alloy was chosen as infiltrating matrix. The optimal conditions for processing of both materials are known, and the aim of this article lays in a description of infiltration success and boundary interaction. The centrifugal casting process offers the feasibility of infiltration not only into open scaffolds but also into those closed by bulk material on one side. This may be the case for Ti-based implants with a bulk centre and porous surface. For comparison, vacuum suction was performed. This process enables infiltration only of open scaffolds. In this case, the pure Zn was chosen for infiltration to prove the negligible effect of minor elements (Al, Cu) on the matrix–reinforcement join.

## 2. Materials and Methods

### 2.1. Sample Preparation

**Figure 1:**
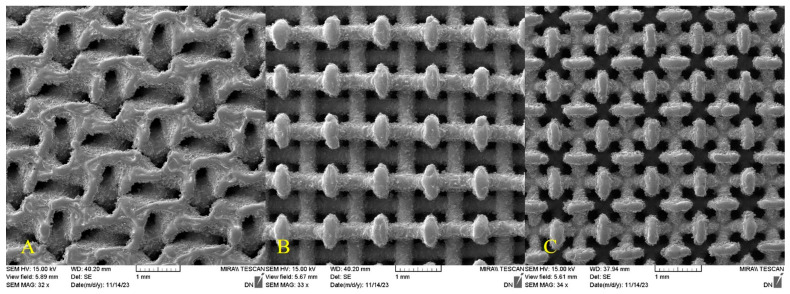
Detail of the used samples structures—scaffold types (**A**) gyroid, (**B**) diamond large, (**C**) diamond small.

Scaffolds used in this experiment were made from Ti-6Al-4V alloy. The condition of their preparation by the SLM method is given in [21]. Three different structure types of scaffolds were used for the experiments, as seen in Figure 1. These all consisted of two parts—porous lattice of various geometry and a solid back (not visible in Figure 1). The solid part was cut off in case some samples to improve the melt flow. Two methods of infiltration were tested—vacuum suction of the melt by vacuum pump and centrifugal casting. An overview of the samples and how they were prepared is summarized in Table 1.

As documented in Table 1, samples 1 and 2 and samples 5 and 6 are equivalent.

### 2.2. Vacuum Pump Suction

**Figure 2:**
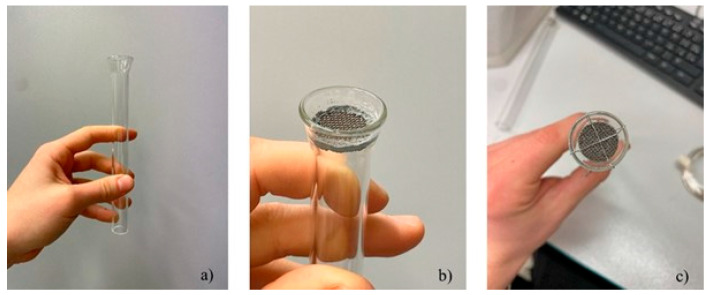
Vacuum suction method: (**a**) glass connecting piece, (**b**) sample attached with glue, (**c**) attached with wire.

Samples 1–4 were made using the vacuum pump and technically pure zinc melt. The 3D-printed Ti-4Al-6V scaffolds without the solid backs were attached to the pump by connecting a piece made from glass, as seen in the Figure 2. To this glass piece, the scaffolds were attached either with the use of the fire-resistant silicate-based glue Technicqll or using a fine silver wire with copper filling. Suction was conducted from a melt temperature 480 °C, and the melt was cleaned by pouring into another crucible before infiltrating samples 3 and 4.

### 2.3. Centrifugal Casting

Samples 5–9 were prepared by centrifugal casting. In these experiments, the Zn-4Al-3Cu was used as a typical Zn-based casting alloy. The 3D-printed scaffolds were placed into rubber mold and subsequently the mold was vulcanized in the automatic vulcanization press PVM 350 CE at temperature of 150 °C, pressure of 150 bar for dwell time of 90 min. In contrast to common centrifugal casting, the scaffolds were let in the rubber mold for the infiltration process. The Zn-4Al-3Al alloy was molten in the F 50 CE furnace and the melt was kept at temperature of 480 °C. The molten alloy was transferred in a crucible to the centrifugal casting device ECO 350 CE, where the vulcanized mold with the scaffold was already rotated at 850 rpm. The outcome of several effects was studied—firstly, the position of scaffolds in the mold during casting (vertical/horizontal direction in relation to the axis of rotation); secondly, the effect of cutting off the solid parts of scaffolds, as given in Table 1.

### 2.4. Microstructure

The microstructure of prepared samples was investigated using an optical microscope Nikon Eclipse MA200 (Tokyo, Japan), a stereomicroscope Olympus SZX10 (Tokyo, Japan) and a scanning electron microscope TESCAN VEGA3 LMU (Brno, Czech Republic) with built-in EDS detector (Oxford Instruments, Oxford, UK). Samples were ground on silicon carbide papers (P400–P4000), polished with D2 diamond paste and a colloidal sillica suspension—either with Eposil F (for samples 1–4), or Eposil M-11 (samples 5–9). Samples 1–6 were etched for the microstructure visualization with Kroll’s etchant (5 mL HNO_3_, 10 mL HF, 85 mL H_2_O), samples 7–9 were etched with the same etchant, but diluted 1:3 with distilled water.

### 2.5. Mechanical Properties Testing

The compression test method was used to observe the mechanical properties of fully infiltrated samples. From these, 2 mm cubes were cut out and tested by the LabTest 5.250SP1-VM (Opava, Czech republic) universal testing machine, at deformation rate of 0.1 mm/min at room temperature. Mechanical properties of the samples, such as yield stress, ultimate stress and elastic modulus, were measured.

## 3. Results and Discussion

### 3.1. Infiltration Success Rate

**Figure 3:**
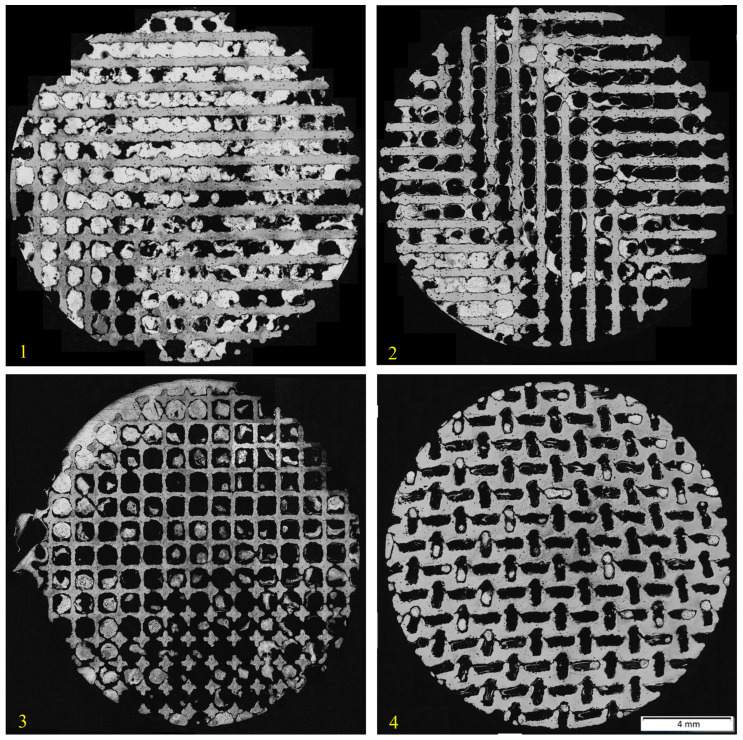
Samples 1–4, prepared by vacuum suction method.

The method of vacuum suction was overall not fully successful. The outcome of the method was uneven and unregularly infiltrated samples, as we can see in Figure 3.

**Figure 4:**
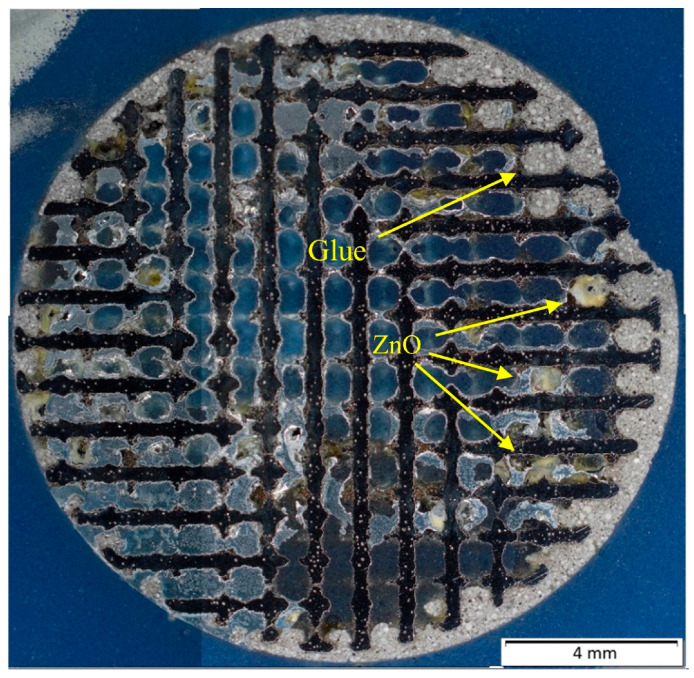
Fire-resistant glue and zinc oxide in sample 2.

In case of samples 1 and 2, quite a big part of the area was filled with the fire-resistant glue, which can be seen in Figure 4. Unfortunately, the samples 3 and 4 were not infiltrated significantly more. In addition, these samples had lower infiltration success rate, most probably due to the geometry of their lattices. The highest infiltration success rate was reached with sample 1, the rate was 53.47%.

**Figure 5:**
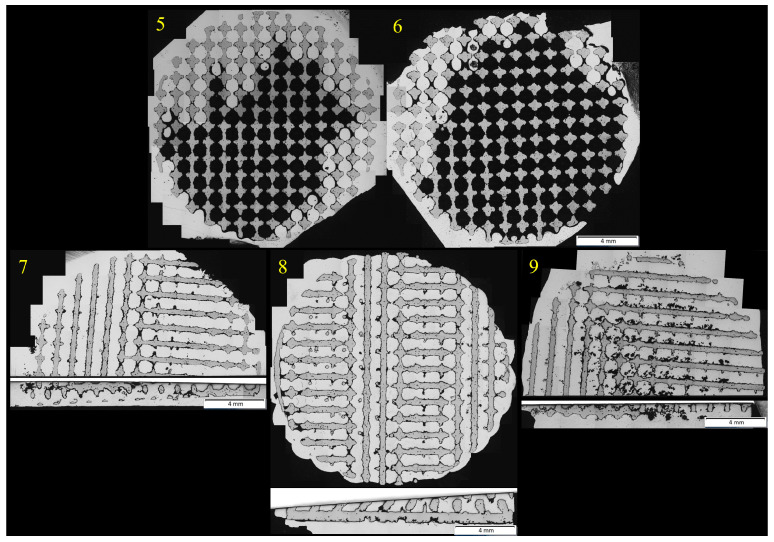
Samples 5–9, prepared by centrifugal casting.

The centrifugal casting method granted more infiltrated samples. All of them are shown in Figure 5. Samples positioned in a vertical direction in relation to the axis of rotation were fully infiltrated, regardless of the presence of the solid back part of the samples. By contrast, the results of horizontally positioned samples varied, depending on the presence of the solid back parts. Sample 7, which had its back cut off, was fully penetrated, unlike the same positioned samples 5 and 6, which had their solid backs (their infiltration success rate was 41.24% and 24.60%). Vertical axis centrifugal casting method seems to be the most suitable method, because it does not need porous material only to fully infiltrate—both sample 8 and 9 were fully infiltrated. This makes it suitable for practice, where it is expected to produce an implant with porous structure on the surface and a non-porous core.

### 3.2. Microstructure

**Figure 6:**
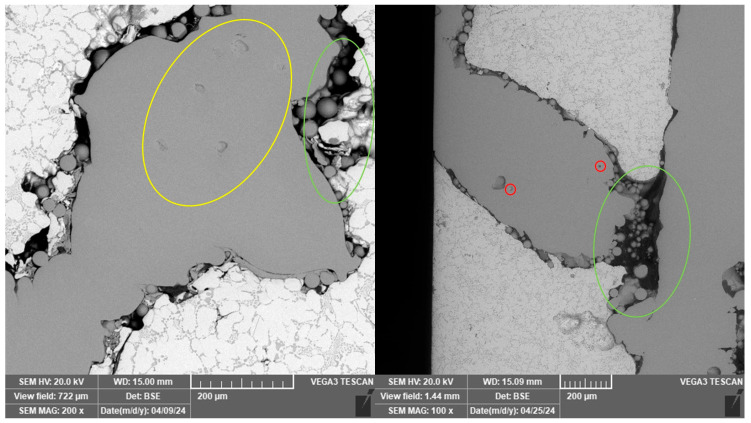
Porosity effects visible in the scaffolds. Gas pores are marked with red, keyholes with yellow and areas with lack of fusion with green.

Porosity effects, typical for additive manufacturing, were observed on all Ti-6Al-4V reinforcements. That includes areas of balling phenomenon and lack of fusion (green), and gas (round, red) and keyhole pores (yellow). These can be all seen in Figure 6.

**Figure 7:**
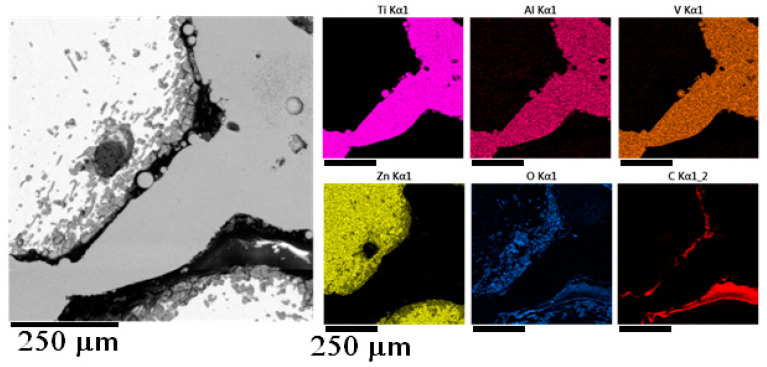
EDS element map of the detail of sample 3.

Samples prepared by the vacuum suction were quite similar to each other in the matter of microstructure. Quite a considerable amount of oxidic inclusions was observed in the microstructure of all samples, even in samples where the melt was cleaned before the infiltration. Zinc oxides were mainly found at the contact surfaces with the titanium reinforcement, which can be seen in the EDS map in Figure 7.

**Figure 8:**
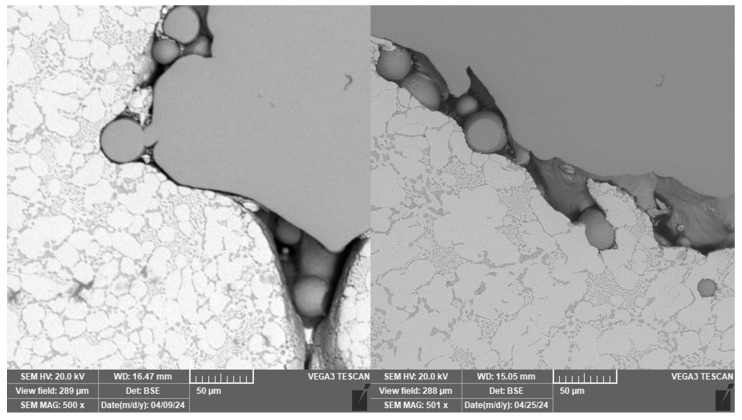
Vertical and horizontal section of sample 8.

Typical polyphase microstructure of the Zn-4Al-3Cu alloy was observed on samples made by centrifugal casting. The microstructure of the samples examined in the vertical section did not differ significantly from the microstructure in the horizontal section (Figure 8). The melt was apparently not sufficiently purified before casting, and so oxide inclusions are also observed in these samples.

**Figure 9:**
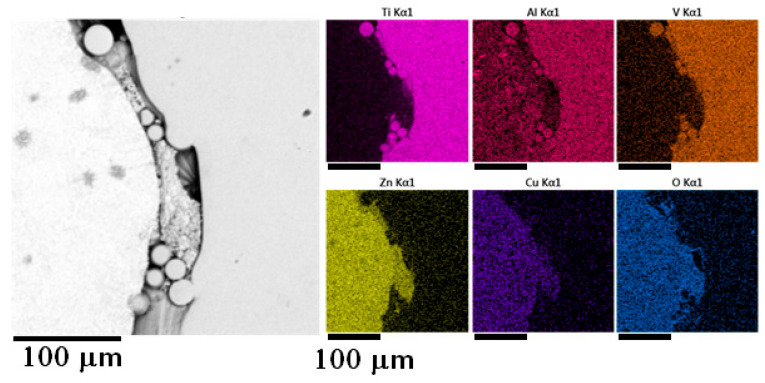
EDS element map of the fine structure of sample 6.

Samples 5 and 6 were significant because of the different microstructure of the zinc alloy near the contact surfaces with the titanium reinforcement. EDS elemental mapping in this area does not indicate the formation of intermetallics, as seen in Figure 9. In between the titanium balls on the surface of the reinforcement, the melt probably cooled more quickly and thus formed a finer structure. In the microstructure of sample 7, this finer structure was not observed. In addition, the EDS linescan was performed at the point of direct contact between the matrix and the reinforcement, which did not show the presence of a brittle intermetallic phase.

**Figure 10:**
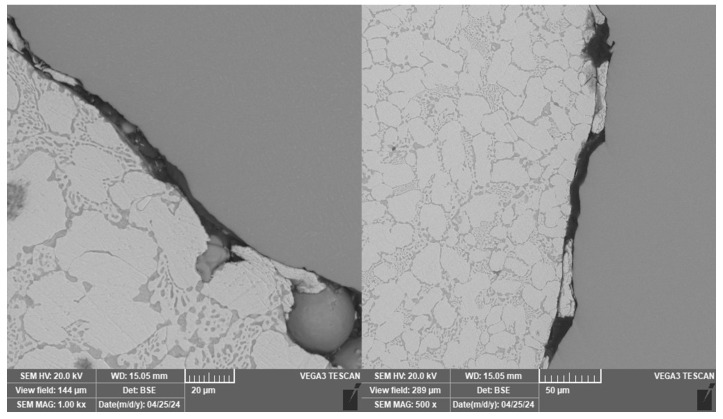
Separated thin bands of the matrix on sample 8.

In Figure 10, thin bands of zinc alloy around the titanium reinforcement, separated from the surrounding material, observed on the sample 8, are visible.

**Figure 11:**
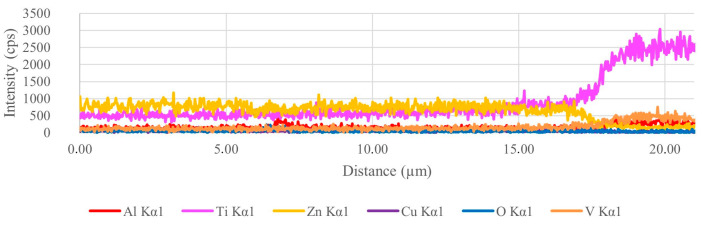
EDS linescan of sample 8.

The EDS linescan (Figure 11), taken at the interface between the unstripped matrix and the reinforcement, does not indicate the presence of an intermetallic phase, and the microstructure of the matrix fragments remaining here is not significantly different from the rest of the matrix. The contact of the zinc melt with the titanium reinforcement was probably so rapid that the intermetallics did not have time to form.

**Figure 12:**
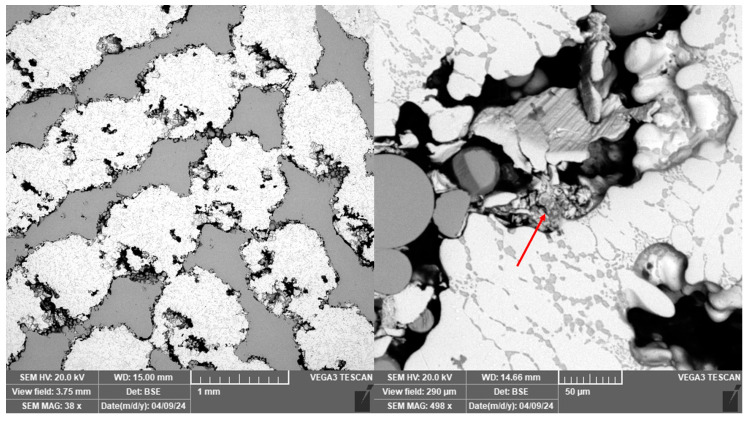
Traces of inclusions in sample 9.

For sample 9, higher porosity was observed despite full infiltration than for the other samples prepared by centrifugal casting. The melt was apparently not perfectly cleaned. The oxide inclusions were most likely pulled out of the sample structure during the surface treatment process to leave behind specifically shaped pores (Figure 12). Residual oxides are labelled with red arrow.

**Figure 13:**
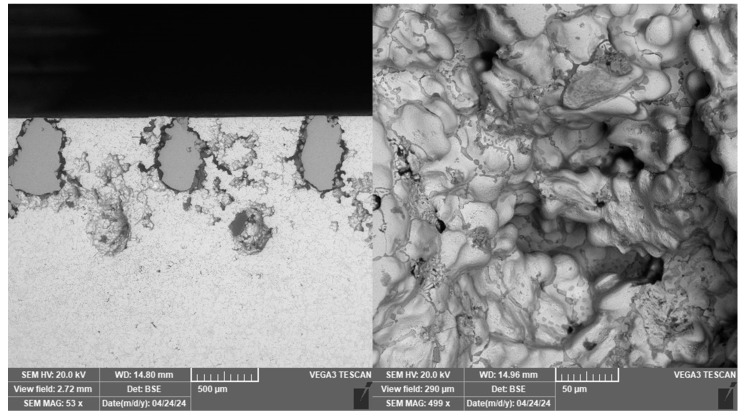
Specifically shaped pores after removal of the reinforcement on sample 9.

Traces of pulled out incoherent reinforcement sections were also observed in the vertical section through the sample. These, unlike the inclusion marks, are regularly spaced and rounded, as observed in Figure 13. The incoherence of the material in this case is probably due to insufficient fusion of the material during the manufacture of the reinforcement.

The microstructure is incomparable with TiAlV/Zn composite prepared by friction stir process described in [19] because that microstructure is significantly finer with residual stresses. It is possible to observe similarities in microstructure of samples of steel 3D printed scaffolds infiltrated by aluminium alloy prepared by centrifugal casting [22]. The authors distinguished intimate and nonintimate interfaces between matrix and reinforcement. Most interfaces presented in this paper are nonintimate due to the presence of powder particles on the 3D-printed scaffold surface. It was proven in this study that the temperature of 480 °C is sufficiently high for successful infiltration of Zn-based melt, but this temperature is not hight enough for creation of intermetallic phases at the interface, which happened at higher temperatures [23,24].

### 3.3. Uniaxial Compression Tests

**Figure 14:**
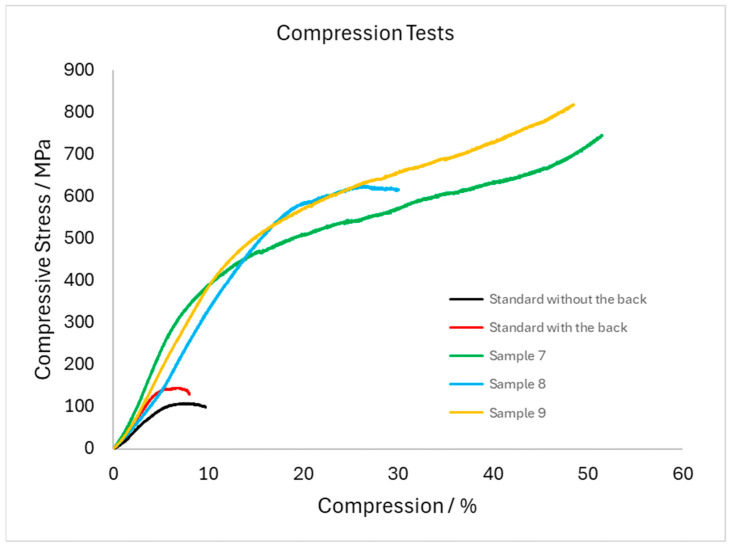
Compression test curves—example for each sample type.

The results of uniaxial compression tests are highly distorted and scattered due to the non-standard dimensions of the samples and the geometry of the scaffolds. Therefore, it is not possible to compare them with the data from the literature (see the first two rows of Table 2), only to compare the test result of the specimen and the corresponding uninfiltrated scaffold standard. The typical compression curves are given in Figure 14. The initial uninfiltrated samples taken as a standard were cut (i) fully in the porous part (labelled as standard without the back) or (ii) at the boundary between porous scaffold and bulk (labelled as standard with the back). Their comparison illustrates the influence of porosity fraction on mechanical properties and as expected, the sample with mixed (porous and bulk) structure reached higher values of compressive stress. For samples 7 (infiltrated in horizontal position) and 8 (infiltrated in vertical position), the standard is without the back, non-porous part, for sample 9 (infiltrated in vertical position) it is the standard with the back. However, even from these very scattered results, an improvement in the mechanical properties of the zinc alloy infiltrated specimens compared to the uninfiltrated scaffold standards can be observed, especially in terms of yield strength and ultimate strength.

Mechanical properties of 3D-printed scaffolds (standards) were significantly different from infiltrated samples (samples 7–9). It contrasts with samples studied in [20], where the mechanical properties are in the same order of magnitude.

## 4. Conclusions

TiAlV/ZnAlCu composite materials were successfully prepared using 3D-printed Ti-6Al-4V scaffolds that were infiltrated by Zn-4Al-3Cu molten alloy by centrifugal casting. The most typical Ti-based alloy was chosen for 3D printing as well as the typical Zn-based casting alloy for infiltration. Results presented in this paper prove the feasibility of infiltration process. It is promising starting point for future study with biocompatible materials. The infiltration process was successful for vertical and also for horizontal (parallel and perpendicularly to the built-up direction of 3D printing) position of the scaffold in the casting rubber mold. With this method in vertical arrangement, fully infiltrated material was prepared, independently of the presence of the solid back part. This enables the preparation of composite with Ti-based core covered by composite material. Moreover, the solidification of the melt in this process was so quick that the intermetallic phase did not have time to form. These aspects make this method suitable for practice, where it is suggested to use it to infiltrate a biomaterial with porous lattice on the surface and a solid core.

The simplified infiltration method by vacuum suction was evaluated as non-suitable, because it provides non-fully-penetrated material with unpredictable infiltration rate.

## Figures and Tables

**Table 1:** Overview of the samples.

Sample Number	Lattice Structure	Solid Back	Method	Notes
1	B	×	vacuum suction	attached with glue
2	B	×	vacuum suction	attached with glue
3	C	×	vacuum suction	attached with wire
4	A	×	vacuum suction	attached with wire
5	B	✔	centrifugal casting	horizontal position
6	B	✔	centrifugal casting	horizontal position
7	B	×	centrifugal casting	horizontal position
8	B	×	centrifugal casting	vertical position
9	B	✔	centrifugal casting	vertical position

**Table 2:** Results of the uniaxial compression tests form Figure 14 (average of 3 measurements).

Sample	Rp0.2 (MPa)	Rm (MPa)	Elastic Modulus (MPa)
Ti-6Al-4V (bulk)	848	1080	[25]
Zn	75	160	[26]
Standard without the back	75 ± 17	123 ± 11	27 ± 3
Standard with the back	124 ± 23	168 ± 9	42 ± 3
Sample 7	287 ± 23	556 ± 38	57 ± 2
Sample 8	421 ± 29	669 ± 21	49 ± 5
Sample 9	332 ± 55	539 ± 35	46 ± 2

## Data Availability

The data used are available under the Zenodo link: https://doi.org/10.5281/zenodo.16894451.
